# High-Quality Application of Crayfish Muscle in Surimi Gels: Fortification of Blended Gels by Transglutaminase

**DOI:** 10.3390/gels11030204

**Published:** 2025-03-14

**Authors:** Hongyi Wang, Qiang Li, Mengru Yang, Hong Wang, Mengtao Wang, Lin Lin, Jianfeng Lu

**Affiliations:** 1 Engineering Research Center of Bio-Process, Ministry of Education, School of Food and Biological Engineering, Hefei University of Technology, Hefei 230009, China; wanghongyi.hfut@gmail.com (H.W.); lq00742021@163.com (Q.L.); 2023111323@mail.hfut.edu.cn (M.Y.); 13505612025@163.com (H.W.); wmt13027660032@163.com (M.W.); 2 Anhui Province Key Laboratory for Agriculture Products Modern Processing, School of Food and Biological Engineering, Hefei University of Technology, Hefei 230009, China

**Keywords:** surimi, transglutaminase, crayfish muscle, blended gel, gel properties

## Abstract

The application of crayfish muscle in surimi products is a potential way to promote their processing and ensure that it is of a high value. In this study, a one-way completely randomized design was used to prepare mixed surimi gels with different proportions of crayfish muscle. The effect of transglutaminase (TGase) on the improvement in the structural properties, water-binding capacity, micromorphology and protein conformation of blended gels was explored using mass spectrometry, centrifugation, scanning electron microscopy, and Fourier transform infrared spectroscopy. The results of thus study were analyzed by one-way ANOVA showed that in the absence of TGase, crayfish muscle made the microstructure of the blended gel looser and rougher, with a reduction in the strength of the gel and a decrease in the water holding capacity. The addition of 0.6% TGase was able to ameliorate this negative effect by promoting the formation of key chemical bonds and changes in protein conformation, which ultimately led to the enhancement of the crayfish–surimi blended gel properties. Practically, this study provides a viable strategy for incorporating crayfish into surimi products, enabling the development of novel, high-quality seafood products with improved texture and moisture retention, thereby enhancing consumer appeal and reducing waste in crayfish processing.

## 1. Introduction

Crayfish (*Procambarus clarkii*) are native to northeastern Mexico and the South Central United States, and subsequently, their distribution has gradually expanded to almost the entire globe, with the exception of Antarctica and Oceania [1]. Crayfish have a strong ability to adapt to the environment and are an important economic freshwater product in China, with their aquaculture production reaching 3,161,000 tons in 2023 alone, an increase of 9.35% over that in the previous year [2]. In addition, crayfish also has a delicious flavor, nutritional value and high food value, and very good processing and utilization prospects [3]. In 2023, 1,402,300 tons of crayfish was processed in China, making up 44.36% of farmed production [2]. However, at present, processed crayfish are mainly distributed as fresh crayfish and single frozen crayfish (whole crayfish and crayfish tails). Serious homogenization of crayfish processing products limits the high-quality development of the crayfish processing industry.

The preparation of surimi gels shows high compatibility with a wide range of food-borne ingredients. The application of protein-based low-value ingredients to the development of surimi products to enhance their application is a widely used approach, and a large number of studies have been devoted to it. These proteins contain both plant and animal sources. Plant proteins, as a low-cost and widely available food processing ingredient, have received equal attention for their application in surimi gels. Different plant proteins can participate in hydrophobic interactions, disulfide bond formation, and protein structural rearrangements to couple with myofibrillar proteins in surimi to form blended gels [4]. Further enhancement of the addition of plant proteins could also lead to the development of softened surimi gels with higher in vitro digestibility [5]. Research related to low-value raw materials of animal origin in surimi gels is equally extensive. Chinese mitten crab meat was applied to the preparation of surimi gel in an amount of up to 10% of the gel [6]. Based on microwave puffing technology, surimi gels containing crab meat exhibiting degree of fibrillation of up to 2.23% have also been derived [7]. Animal-derived protein raw materials such as chicken breast, egg, and plasma are also thought to be able to participate in the formation of the gel matrix and are used in the preparation of surimi gels [8,9,10]. In addition, some protein hydrolysis products have strong functional properties due to them carrying a large number of surface active groups, and they often show good application in surimi gels [11,12]. In summary, the application of crayfish muscle in the preparation of surimi blended gels is a promising development strategy. It is not only conducive to solving the problem of the huge production of crayfish using a single form of processing, but also conducive to encouraging surimi processing enterprises to develop new high-value-added products. Therefore, the development of crayfish surimi blended gel is not only a technological breakthrough, but also an important strategy to promote industrial transformation and respond to the trend of efficient resource utilization and improvements in consumption.

The quality of surimi gels is mainly reflected by its gel properties. For this reason, rinsing is usually required during the preparation of surimi to remove components that are not conducive to gel formation, such as proteases and water-soluble proteins with poor gelation properties [13]. These components are also present in crayfish muscle, and the complexity of its composition also reduces the relative concentration of the main component in gel formation (myofibrillar protein). These factors could potentially severely limit the gel properties of surimi gels blended with crayfish. Currently, a large number of studies have been devoted to the enhancement of the gel properties of surimi. In this regard, some novel processing techniques have been introduced into the gelatinization process of surimi. Microwave heating can utilize the dielectric effect of surimi to increase the gel strength of low-salt surimi gels to 821.74 g·cm by promoting protein solubilization and cross-linking [14]. The mechanical effect and cavitation generated by ultrasonic treatment can promote the unfolding of structural proteins and expose more hydrophobic groups and reaction sites, thus enhancing cross-linking between protein molecules in surimi [15]. Appropriate radiofrequency heating induces the conversion of α-helixes into random curls and promotes hydrophobic interactions between and the cross-linking of proteins by disulfide bonds to improve the structural properties of grass carp protein gels [16]. In addition, ohmic heating is also an emerging method for celiac gel fortification [17]. The enhancement of surimi gel by exogenous additives is more stable and simple to implement compared to the physical field technique. Polysaccharide additives enhance the structural properties of surimi gels through the synergistic effects of matrix enhancement, water binding and encapsulation, and covalent–noncovalent interactions [18]. The phase behavior of polysaccharides during the gelatinization of surimi can also be used to judge their appropriate concentration to improve the physicochemical properties of blended gels [19]. TGase catalyzes the generation of ε-(γ-Gln)-Lys bonds from ε-amino group with the γ-carboxy amide group in glutamine and promotes intermyosin cross-linking [20]. Therefore, it is considered to be an effective fortifier for surimi gels. TGase could enhance the surimi blended gel properties of a variety of exogenous proteins intervening in surimi, including chickpea protein, sheep plasma protein, and crabmeat, to name a few [6,21,22]. Even in different gelation environments, including intrinsic environments such as salt ion concentration and extrinsic environments such as processing methods, TGase was able to promote cross-linking between proteins in the blended system [21,23]. The broad adaptability of TGase opens up possibilities for the use of crayfish muscle in surimi gels. However, it has not been clarified whether or not the lifting effect of TGase on surimi blended gels is affected by the type and concentration of ingredients other than surimi; it is also unclear what the coupling characteristics of crayfish muscle in this catalytic effect are. Related research will facilitate the high-quality application of crayfish muscle in surimi gels and promote the industrial innovation of crayfish and surimi products.

In this study, surimi-based blended gels containing crayfish muscle were investigated. The effects of different crayfish muscle additions (0%, 2.5%, 5%, 7.5%, and 10%) on the gel properties in the presence or absence of TGase were investigated by analyzing the textural features, water binding capacity, micro-morphology, and protein conformation of the blended gels. The results of this study may provide theoretical references for the high-value utilization of crayfish muscle in surimi products, to promote the utilization of crayfish in deep processing, and to alleviate the problem of homogenization of processed crayfish products.

## 2. Results and Discussion

### 2.1. Effect of TGase and Crayfish Meat on the Texture Properties of Blended Gels

#### 2.1.1. Texture Properties

**Figure 1:**
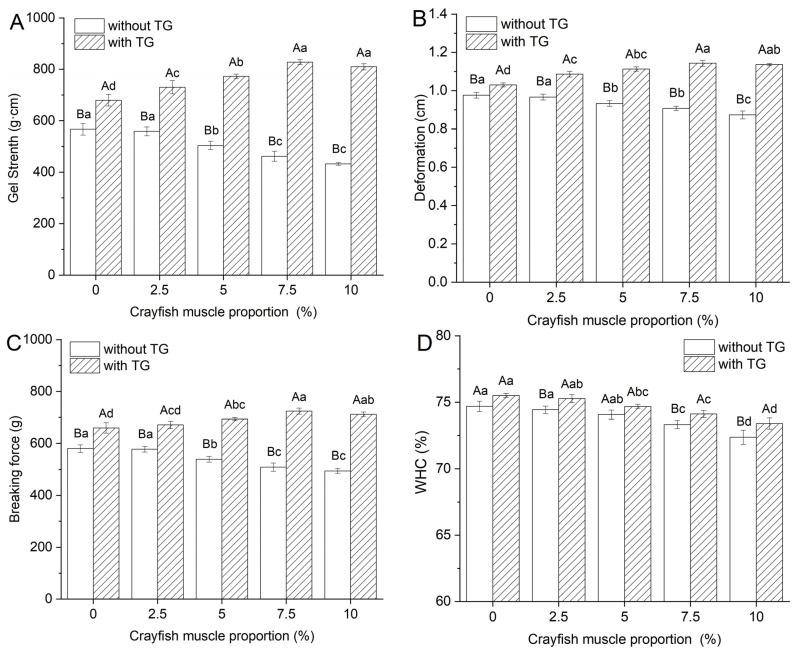
Changes in breaking force (**A**), deformation (**B**), gel strength (**C**), and WHC (**D**) of blended gels. Lowercase letters above each standard deviation bar reveal significant variations among gels with different crayfish muscle contents (*p* < 0.05). Uppercase letters highlight significant differences between samples with and without added TGase (*p* < 0.05).

Figure 1A–C show the effect of different amounts of crayfish muscle on the breaking force, deformation and gel strength of surimi blended gels, and the intervention of TGase on this effect. A decreasing trend in gel strength and breaking force in gels without TGase was observed as the crayfish muscle content increased. Notably, with 5% inclusion of crayfish muscle, significant reductions in breaking force, deformation, and gel strength were documented, dropping from 580.28 g, 0.98 cm, and 566.89 g·cm to 539.64 g, 0.93 cm, and 503.72 g·cm, respectively (*p* < 0.05). These trends are similar to those found in previous studies for the addition of Chinese mitten crab meat to surimi gels; this may be due to the co-precipitation of myoplasmic proteins with myofibrillar proteins in the added crayfish, which weakened the structure of the gel [6,24]. Interestingly, the TGase-containing gels improved in all measured gel strength characteristics after the addition of moderate amounts of crayfish muscle, highlighting the positive impact of TGase on gel properties. Research indicates that TGase’s role in forming ε-(γ-Glu)-Lys covalent bonds stabilizes the protein structure within the gel, substantially boosting gel strength [25]. The addition of microbial transglutaminase (MTG) has been found to improve gel properties notably [26]. As crayfish muscle content increased up to 7.5%, the differences in gel properties between TGase-added and non-TGase-added gels became more pronounced, though no further differences were seen between the 7.5% and 10% groups.

#### 2.1.2. Effect of TGase and Crayfish Muscle on the Water Holding Capacity of Blended Gels

Water holding capacity (WHC) is an indication of the ability of crayfish–surimi blended gels to bind to water, which can reflect the stability of the gel system laterally [27]. The effect of crayfish muscle and TGase addition on the WHC of the blended gel is shown in Figure 1D. The WHC of the blended gels decreased with increasing crayfish muscle content, with or without the addition of TGase. This decline was significant when the amount of crayfish muscle exceeded 7.5%. This reduction in WHC may be linked to the structural properties of crayfish proteins and their interactions within the gel matrix. However, at the same level of crayfish muscle addition, the blended gels containing TGase consistently showed higher WHC, suggesting that TGase improves the water holding capacity of these gels. This enhancement is attributed to the role of TGase in the promotion of myofibrillar proteins’ cross-linking reactions, forming a denser gel network that effectively retains more water [28]. Therefore, TGase can effectively enhance the water retention capacity of crayfish–surimi blended gels and stabilize the gel system.

### 2.2. Effect of TGase and Crayfish Muscle on the Color Properties of Blended Gels

**Figure 2:**
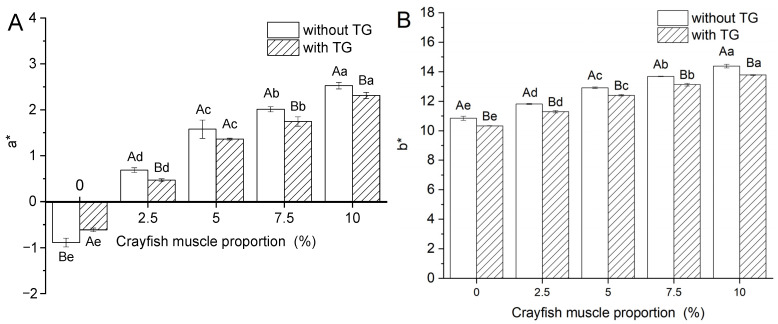

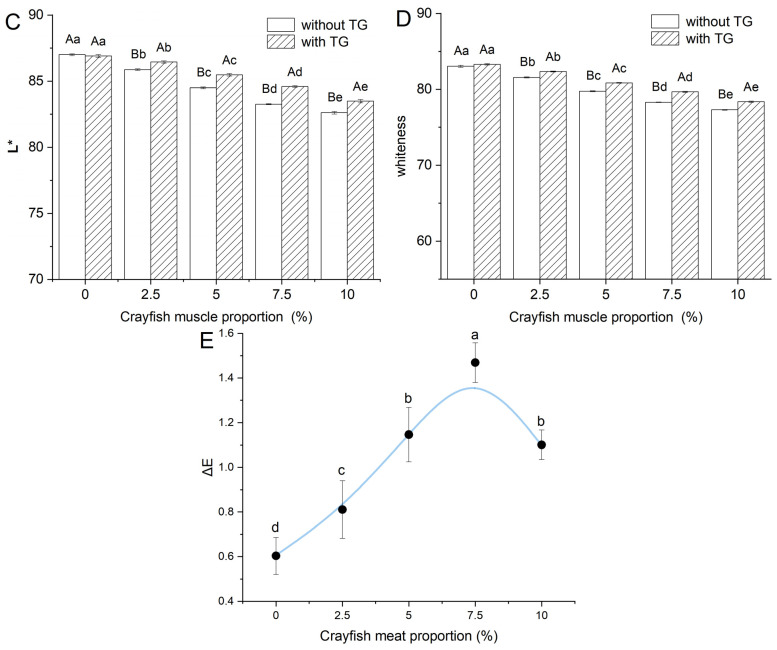
Changes in a* (**A**), b* (**B**), L* (**C**), whiteness (**D**) and ΔE (**E**) of blended gels. Lowercase letters above each standard deviation bar reveal significant variations among gels with different crayfish muscle contents (*p* < 0.05). Uppercase letters highlight significant differences between samples with and without added TGase (*p* < 0.05).

The magnitude of the whiteness value is often used as an indication of the tightness of the surimi gel structure; when the texture is tight, there is a greater ability to reflect light, which will exhibit a larger whiteness value. This is calculated from the red–green value (a*), the yellow–blue value (b*) and the luminance value (L*). Figure 2 illustrates the effect of crayfish muscle and TGase addition on each of the color characteristics of the surimi blended gels. The increase in the amount of crayfish muscle significantly affected the color properties. The increase in a* and b* may be attributed to the presence of blue and red carotenoproteins and the oxygen-carrying molecule, hemocyanin, found in crayfish [29]. On the other hand, the reduction in gel strength implies that the crayfish muscle may have disrupted the homogeneity of the texture of the surimi blended gels, resulting in reduced light reflectivity on the gel surface and poorer L* and whiteness [6]. The application of TGase did not notably affect the whiteness of surimi gels that lacked crayfish muscle (*p* > 0.05). However, at the same level of crayfish muscle addition, the surimi blended gel containing TGase showed lower a* and b* values as well as higher L* and whiteness (*p* < 0.05). This indicates that TGase contributes to the development of a denser gel network, which enhances the gel’s ability to reflect visible light, thereby improving its whiteness. The enhancement of the whiteness of surimi gels by TGase is a more general phenomenon, even in different thermal gelation methods [23]. This phenomenon is consistent with the higher performance of gel strength and WHC. A comprehensive observation of the color characteristics of the crayfish–surimi blended gel (Figure 2E) showed that the effect of TGase on the overall color characteristics of the blended gel was greatest (*p* < 0.05) when the crayfish muscle addition was 7.5%. This was consistent with the fact that its gel strength was no longer significant (*p* > 0.05) after increasing to 827.79 g·mm. Overall, crayfish muscle may lead to a reduction in the color properties of surimi hybrid gels due to a number of reasons, including compositional characteristics, and TGase may attenuate this negative effect through cross-linking facilitation.

### 2.3. Effect of TGase and Crayfish Muscle on the Water Distribution of Blended Gels

**Figure 3:**
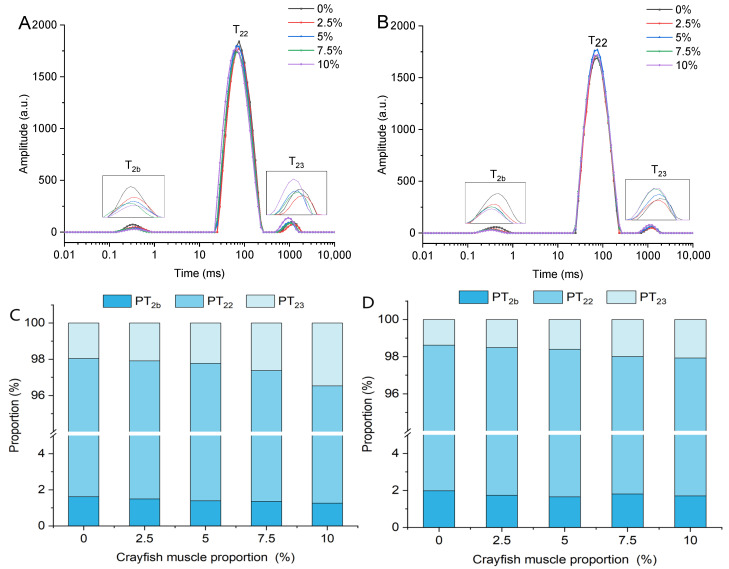
The effect of different treatments on the spin relaxation time (T_2_) of blended gels treated (**A**) without TGase and (**B**) with TGase, as well as the percentage of relative area of peaks (**C**,**D**). Note: PT_2b_, PT_22,_ and PT_23_ represent the peak area ratios of T_2b,_ T_22_, and T_23_, respectively.

The low-field nuclear magnetic resonance (LF-NMR) technique can be used to analyze the rate and proportion of water molecules migrating through a gel by measuring the relaxation properties of the hydrogen atoms in the gel [30]. LF-NMR was applied to investigate the effects of crayfish muscle and TGase addition on the co-water distribution characteristics of surimi blended gels, as shown in Figure 3A,B. The relaxation spectra show three separate peaks, each indicating a distinct water state. T_2b_ (0.1–1 ms) indicates water molecules tightly bound to the gel matrix. T_22_ (20–400 ms) indicates water molecules tightly encapsulated by the gel matrix. T_23_ (400–2000 ms) indicates free water molecules outside the gel matrix. The T_2_ relaxation times directly reflect the mobility of water molecules; longer T_2_ times suggest greater migration capabilities [31]. The T_2b_ relaxation time of the blended gel increased with increasing crayfish muscle addition, while the relaxation times of T_22_ and T_23_ decreased (Figure 3A). This suggests that the addition of crayfish muscle reduced the stability of bound water in the blended gel, while the stability of immobile and free water was improved. Among them, the peak area of T_22_ is clearly larger than the other peak areas, and its representation of water that does not flow easily is also the main observation in the related study. When TGase was added, the T_2b_ relaxation time and T_23_ relaxation time of the blended gels were reduced to a degree that increased with the increase in the amount of crayfish muscle (Figure 3B). This suggests that the addition of TGase made the bound and free water more stable, while the relaxation time of the less mobile water did not change significantly.

The proportions of the three water distributions expressed by the peak area share are shown in Figure 3C,D. As the crayfish muscle content increased, the PT_2b_ of blended gel gradually decreased while PT_23_ gradually increased, suggesting that adding crayfish muscle enhanced the escape capability of bound water in the gel. Figure 3D shows that the addition of TGase altered the relaxation peak area percentages of bound water and free water. Compared to the group without TGase, the PT_23_ of the TGase-added group significantly decreased, indicating a decrease in free water content within the gel following the incorporation of TGase. However, as the content of crayfish muscle increased, PT_23_ in blended gels with TGase continued to show an upward trend. Additionally, after adding TGase, the proportion of bound water generally increased, while the proportion of immobile water exhibited no significant changes. Thus, the application of TGase resulted in an elevation in bound water content coupled with a reduction in the proportion of free water. This effect is due to TGase inhibiting the conversion of bound water to free water in blended gels, resulting in changes in water distribution and enhanced water retention. Similarly, TGase-catalyzed cross-linking between soy protein and scallop muscle was found to be inhibited by the conversion of bound water [32].

### 2.4. Effect of TGase and Crayfish Muscle on the Magnetic Resonance Imaging of Blended Gels

**Figure 4:**
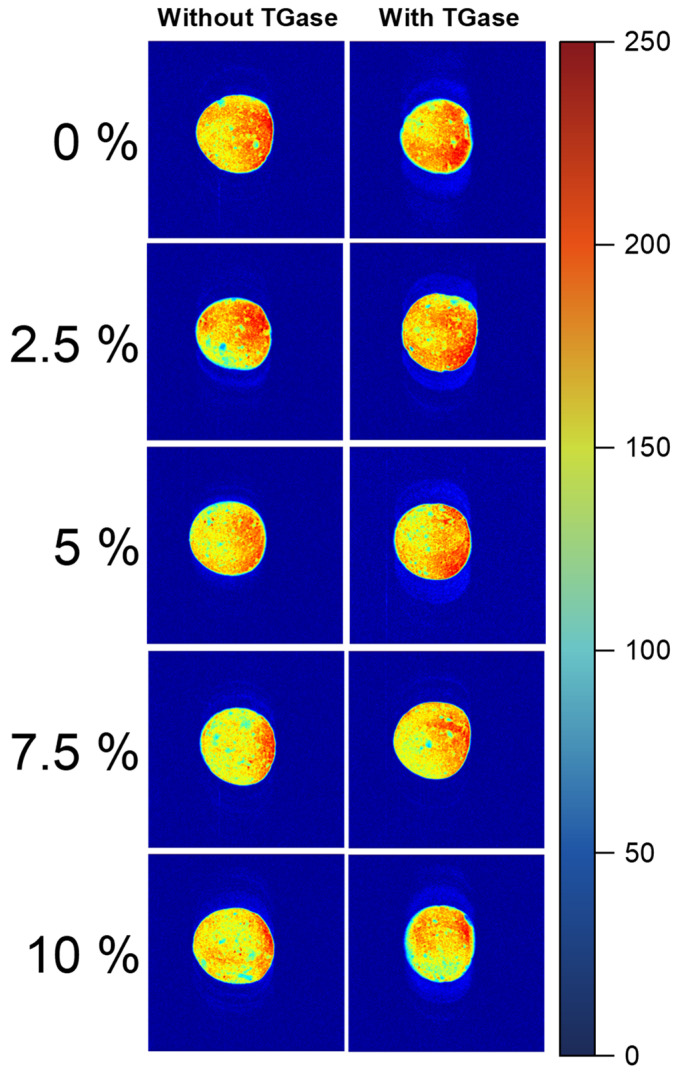
T2-weighted magnetic resonance images of surimi gel with different amounts of crayfish muscle (0%, 2.5%, 5%, 7.5% and 10%) in the samples with TGase and without TGase.

Magnetic resonance imaging (MRI) can efficiently, accurately and non-destructively visualize the state of water distribution in a gel system. The darker-color (tending toward blue) portion of the MRI is meant to reflect a lower distribution of hydrogen protons, indicating the poorer water retention capacity of the gel, and vice versa (brighter color tending toward red) indicates a higher distribution of hydrogen protons and the better water retention capacity of the gel [33]. The effect of crayfish muscle addition and TGase on the hydrogen proton density in surimi blended gels is shown in Figure 4. Variations in these images reveal that with increasing additions of crayfish muscle, the samples exhibit fewer red areas and more yellow areas, indicating an increase in the uneven distribution of water. In addition, under the same level of crayfish muscle addition, a significant increase in red areas and a more homogeneous distribution was observed in the MRI of the blended gel after the introduction of TGase. This enhancement shows that adding TGase improved the bound water retention within the blended gel network, thereby improving the water holding capacity of the crayfish–surimi blended gel. This phenomenon is consistent with the state of moisture distribution and the results of the correlation analysis of WHC. This further validates the negative effect of crayfish muscle on surimi blended gels and the ameliorating effect of TGase on this negative effect.

### 2.5. Effect of TGase and Crayfish Muscle on the Microstructural of Blended Gels

**Figure 5:**
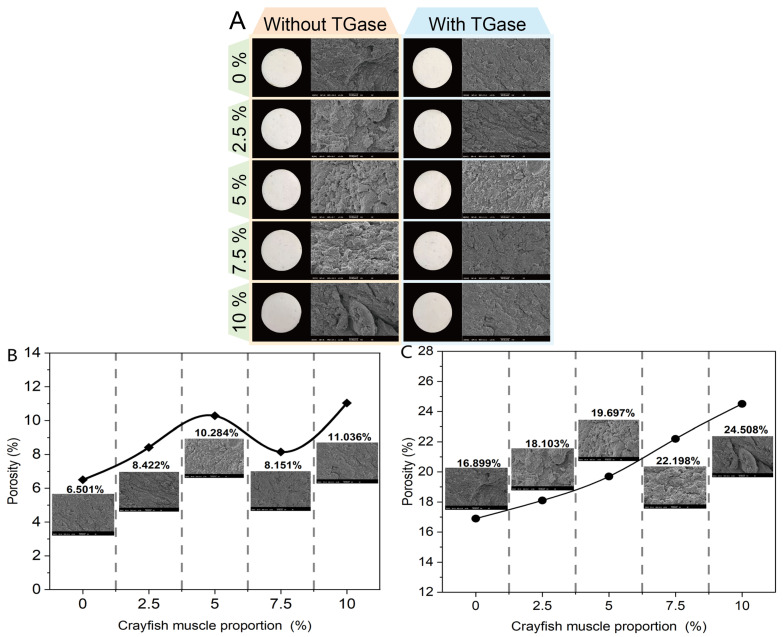
Effect of different crayfish muscle additions (0%, 2.5%, 5%, 7.5%, 10%) and TGase addition on the microstructure (**A**) and porosity ((**B**): with TGase, (**C**): without TGase) of surimi blended gels.

In Figure 5A, the pictures of each group on the left provide a visualization of the untreated crayfish–surimi blended gel. Visual observation cannot not reveal the effect of crayfish addition and TGase addition on the apparent differences in the surimi blended gels, both in terms of structural and color characteristics. Scanning electron microscopy (SEM) is a commonly used method to observe the microstructure of surimi gels [34]. It was able to visualize the extensive effect of crayfish muscle and TGase addition on the structure of the surimi blended gel. In Figure 5A, the images to the right of each group show the results of SEM observations. The results of porosity quantification in the images obtained using image analysis software are shown in Figure 5B,C. As the crayfish muscle content increased, the microstructure of the blended gel became rougher and more irregular, while the porosity increased. The structural damage induced by crayfish muscle inevitably led to a reduction in the water retention capacity of the blended gel, as well as a reduction in gel strength due to stress reduction. Thus, the microstructural observations are consistent with the results of correlation analyses such as that of WHC. The introduction of TGase was more effective in optimizing the microstructure of the blended gels. The addition of TGase reduced the porosity from 24.508% to 11.036% when the crayfish muscle content was 10%. This effect resulted from TGase influencing the protein molecule interactions and the covalent bonding between glutamine and lysine residues, allowing myofibrillar proteins to aggregate more effectively and form a dense protein gel network. According to recent research, reduced porosity enhances the gel’s ability to trap excess moisture, thereby improving its textural properties [35]. The most significant effect of TGase on the reduction in the porosity (8.151%) of the blended gel was observed when crayfish muscle was added at 7.5%. This suggests that the molecular structure was most uniform at this point, with the most significant protein cross-linking, corresponding to the most compact three-dimensional gel network. This finding is consistent with the enhanced gel strength observed in the experiments.

### 2.6. Effect of TGase and Crayfish Muscle on the Chemical Interactions of Blended Gels

Noncovalent bonds, including hydrogen, ionic, and hydrophobic interactions, and disulfide bonds are critical in the formation of surimi gels [36]. The effect of crayfish muscle addition and TGase on the content of chemical interactions in the surimi blended gels is shown in Table 1. In myofibrillar proteins, interactions occur between carbonyl and amino groups within and across peptide chains mainly through hydrogen bonds [37]. According to Table 1, there is a notable decrease in hydrogen bonds in blended gels as the content of crayfish muscle increases (*p* < 0.05). This decrease may be linked to the lipids in crayfish muscle, which can obstruct the formation of hydrogen bonds, thereby adversely affecting water retention, as corroborated by earlier WHC measurements. This underscores the pivotal role of hydrogen bonds in maintaining water stability within the gel matrix. The addition of TGase appeared to significantly enhance the formation of hydrogen bonds between proteins, possibly due to the rearrangement of hydrogen bonds induced by conformational changes during protein cross-linking [38]. This course of action mitigates the negative effects of crayfish muscle addition on hydrogen bond formation to some extent.

Ionic bonds are crucial in sustaining the tertiary and quaternary structures of myofibrillar proteins. During gel processing, the disruption of these bonds under various conditions facilitates cross-linking reactions, which are essential for forming a robust gel network [26]. Table 1 illustrates that the concentration of ionic bonds increases with the addition of crayfish muscle. This suggests that with the addition of crayfish muscle, the proteins in the blended gel system had more stable structural characteristics before gelation and were less susceptible to change during the thermogenic gelation process [39]. However, the presence of TGase significantly reduced the concentration of ionic bonds. The reason for this change may have been that the catalytic action of TGase consumed positively charged lysine residues while reducing the exposure of neighboring charged residues (e.g., glutamate, aspartate), resulting in a reduced opportunity for ionic bond pairing [20]. Similarly, ionic bonding was reduced in TGase-enhanced pea protein isolate gels [40]. This further corroborates the positive role played by TGase in promoting the intermolecular cross-linking of proteins.

Regarding hydrophobic interactions, these are essential for a gel’s structural integrity and are closely linked to gel strength. In samples with TGase, an increase in crayfish muscle content resulted in enhanced hydrophobic interactions, with no significant difference observed between 7.5% and 10% crayfish content. This may be due to the fact that TGase-induced noncovalent cross-linking shortens the spacing of intermolecular hydrophobic groups, making hydrophobic interactions easier to form [41]. At the same time, the formation of hydrophobic interactions further enhances the intermolecular cross-linking. This process of action was similarly found in TGase-containing cowpea protein isolate gels [42]. Thus, at a certain crayfish muscle threshold, the presence of TGase catalyzed the improvement in hydrophobic interactions, which improved the mechanical properties of the gel, consistent with the observed improvement in gel strength.

Clearly, the incorporation of crayfish muscle through chemical bonding does not favor cross-linking between proteins in surimi blended gels. However, the addition of TGase can promote chemical cross-linking between proteins through indirect effects such as the smaller molecular spacing mediated by catalysis.

### 2.7. Effect of TGase and Crayfish Muscle on the Total Sulfhydryl Content of Blended Gels

**Figure 6:**
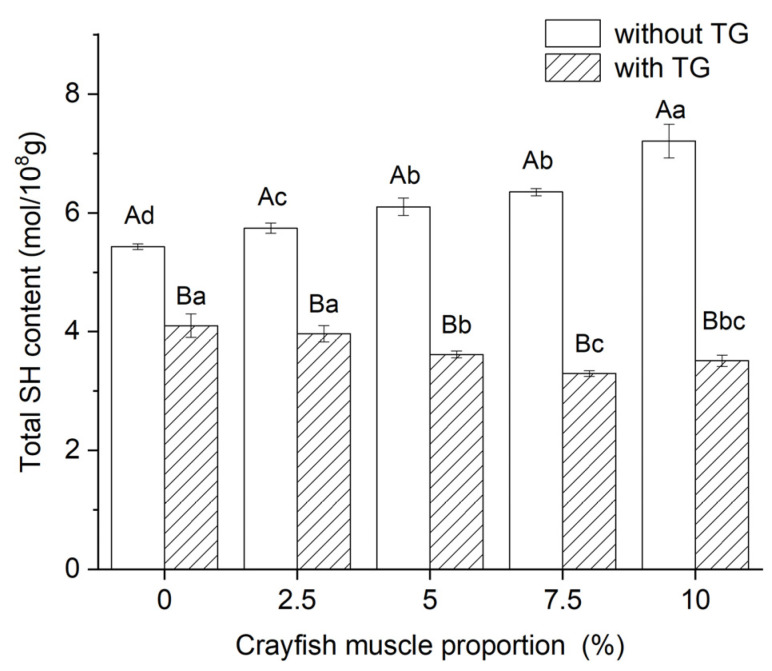
The effect of TG on the total sulfhydryl group content in blended gels. Lowercase letters above each standard deviation bar reveal significant variations among gels with different crayfish muscle contents (*p* < 0.05). Uppercase letters highlight significant differences between samples with and without added TGase (*p* < 0.05).

Sulfhydryl groups are the basis for the formation of disulfide bonds, and disulfide bonds are an important force for stabilizing the structure of surimi gels. The effects of crayfish muscle addition and TGase on the sulfhydryl content in the surimi blended gels is shown in Figure 6. As the muscle content of crayfish increased, the sulfhydryl content also tended to increase. It was hypothesized that this could be due to the hydrolysis of proteins in the blended system into small peptides containing free sulfhydryl groups due to the endogenous proteases (e.g., tissue protease) in crayfish muscle [43]. Although this also implies an increase in the concentration of substrate available for disulfide bond formation, it appears that the amount of disulfide bonds does not increase as a result, and the gel properties of the blended gel are instead diminished. This may be due to the change in potential induced by the changes in the peptide segments introduced by hydrolysis, which nay increase intermolecular electrostatic repulsion and consequently inhibit contact between the sulfhydryl groups [44]. However, a notable difference, as observed when TGase was added to this blended system, was that the sulfhydryl content decreased with increasing crayfish muscle content. This is an indirect indication of the enhanced ability to form disulfide bonds, although protein cross-linking catalyzed directly by TGase is mainly maintained by ε-(γ-Gln)-Lys bonds (non-covalent bonds) and does not involve the formation of disulfide bonds (covalent bonds) [20]. However, the resulting reduction in the spacing between protein molecules promotes contact between sulfhydryl groups, which provides a good precondition for disulfide bond formation [45]. As mentioned earlier, the endogenous protease in crayfish muscle provides more available sulfhydryl groups for the blended system, and the electrostatic repulsion is counteracted by TGase, facilitating binding of sulfhydryl groups to each other to form disulfide bonds. This phenomenon is consistent with the elevating effect of TGase on disulfide bond content in myofibrillar protein gels [45]. Thus, although the addition of small dragon muscle decreases the cross-linking of proteins in the blended system due to its inhibitory effect on disulfide bonds, the catalytic effect of TGase counteracts this phenomenon and allows disulfide bond formation to be promoted.

### 2.8. Effect of TGase and Crayfish Muscle on the Fourier Transform Infrared Spectroscopy of Blended Gels

**Figure 7:**
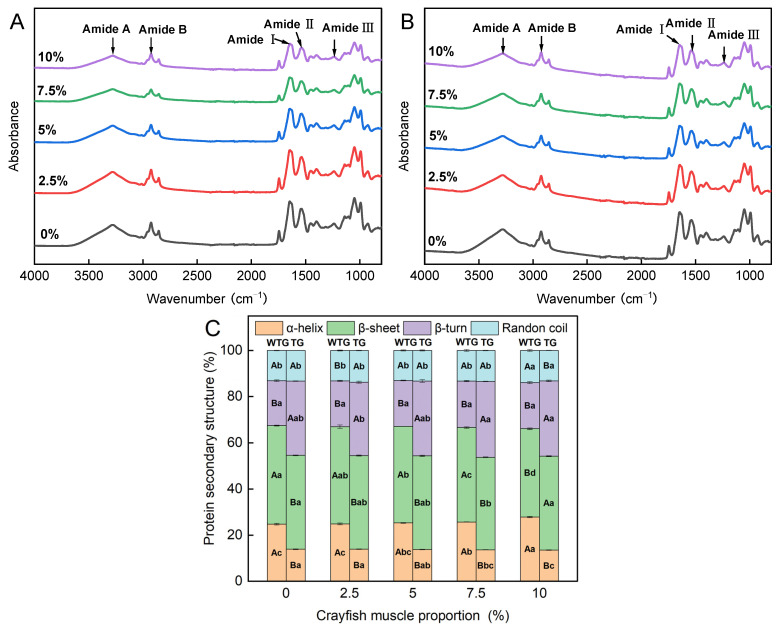
FTIR spectra of surimi gel with varying crayfish muscle contents ((**A**): samples without TGase; (**B**): samples with TGase), and the effect of different crayfish muscle contents on the secondary structure of proteins in the TG and WTG groups of surimi gel (**C**). TG: with TGase; WTG: without TGase. Lowercase letters above each standard deviation bar reveal significant variations among gels with different crayfish muscle contents (*p* < 0.05). Uppercase letters highlight significant differences between samples with and without added TGase (*p* < 0.05).

Fourier transform infrared (FTIR) spectroscopy is commonly used to analyze the composition of protein secondary structures in surimi gels. The FTIR spectra of the crayfish–surimi blended gel are shown in Figure 7A,B. The blended gels had absorption peaks near 1200 cm^−1^, 1500 cm^−1^, 1600–1700 cm^−1^, 2926–2953 cm^−1^, and 3100–3300 cm^−1^, which correspond to the amide III, II, I, B, and A bands of protein molecules, respectively [46,47]. Comparable observations have been reported in cod surimi gels [48]. These wavenumber regions reflect the vibrational modes of various chemical bonds within the protein molecules, providing critical insights into the secondary and tertiary protein structures. As the content of crayfish muscle increases, the peak values of amide A, amide I, and amide II bands tend to decrease. This trend suggests that the addition of crayfish muscle diminishes the hydrogen bonding interactions between protein molecules within the blended gel [49]. Each sample exhibited an absorption peak at 1050 cm^−1^, where the peak intensity decreased as the crayfish muscle content increased. Generally, gel samples subjected to different processing conditions demonstrated analogous spectral graphs, as depicted in Figure 7A,B. All samples featured a peak at 1050 cm^−1^, with decreasing amplitude as the proportion of crayfish muscle increased. Studies on the effect of antifreeze on the protein structure of tilapia showed that the peak near 1050 cm^−1^ was related to the -OH group of sucrose in surimi [50].

The amide I band, typically situated in the 1600 to 1700 cm^−1^ range, corresponds to the vibrational modes of carbonyl (C=O) groups in protein molecules, crucial for revealing the protein’s secondary structure. For α-helices, β-sheets, β-turns, and random coils, distinct absorption peaks are identified within the respective spectral ranges of 1651–1660 cm^−1^, 1600–1639 cm^−1^, 1661–1700 cm^−1^, and 1640–1650 cm^−1^, respectively [51]. As illustrated in Figure 7C, the TGase-added group, compared to the group without TGase, displayed a significant reduction in α-helix content (*p* < 0.05) and a rise in the proportion of β-turns (*p* < 0.05), with minimal effects on random coils and β-sheets. The stability of the α-helix relies on continuous hydrogen bonding within the main chain, and TGase-catalyzed cross-linking may interrupt the arrangement of the original hydrogen bonds, leading to deconvolution of the helical structure [52]. The deconvolution of the helical structure can be enhanced after inverse fold accumulation, which provides a good prerequisite for structural transformation and chemical bond formation [53]. The resulting ordered intermolecular cross-linking of proteins imparts good stress and water retention properties to the blended gels. In contrast, higher α-helix content was thought to reduce the mechanical properties and stability of surimi gels [54]. Thus, while the deteriorating effect of crayfish muscle on surimi blended gels may be attributed to the insufficient transformation of the secondary structure, the addition of TGase was able to enhance the cross-linking between proteins in the blended gels through structural changes by promoting the transition from the α-helix to β-turn. This process of action was demonstrated in microbial the transglutaminase-mediated enhancement of the mechanical properties of pork gels [55].

## 3. Conclusions

This study systematically elucidates the effects of crayfish muscle incorporation and transglutaminase (TGase) addition on the physicochemical properties, microstructure, and molecular interactions of silver carp–surimi blended gels. Increasing crayfish muscle content (0–10%) in surimi gels without TGase progressively reduced gel strength, WHC, and structural homogeneity. This is attributed to the inhibition of hydrogen bonding forcing insufficient structural transformations to occur between proteins and attenuating conformationally mediated cross-linking between proteins. Meanwhile, the inhibitory effect of crayfish muscle on hydrophobic interactions and disulfide bonds in the surimi blended gel further hindered cross-linking between protein molecules. Ultimately, the protein cross-linking in the mixed system is disordered and is reflected by a loose gel structure. Proteases in crayfish muscle have been suggested as potential factors contributing to the latter’s negative effects, and hydrolysis by proteases may alter the peptide composition in the blended system and inhibit protein conformational shifts and the binding of reactive groups through enhanced electrostatic repulsion. The addition of 0.6% TGase counteracted the negative effects of the addition of crayfish muscle. TGase-catalyzed ε-(γ-Gln)-Lys bonds promote protein aggregation while possibly counteracting the electrostatic repulsion induced by crayfish proteases, driving the shift in protein hooking as well as the binding of reactive groups. Under these conditions, protein cross-linking in the blended system becomes more organized. A compact gel structure not only has good water retention capacity, but also has strong stress resistance, which manifests as increased gel strength.

The synergistic use of crayfish muscle (≤7.5%) and TGase represents a viable strategy to develop high-value surimi products with enhanced texture and moisture stability. TGase effectively mitigated the structural defects induced by crayfish muscle; it could thus promote the resource-efficient utilization of underutilized aquatic proteins, and at the same time help to alleviate the problem of product homogenization in crayfish processing.

## 4. Materials and Methods

### 4.1. Materials

The frozen silver carp surimi used in the experiment was AAA-grade (Hubei Honghu Jili Aquatic Food Co., Ltd., Honghu, China). Crayfish were purchased from RT-Mart Supermarket (Hefei, China), and crayfish muscle was taken by hand by removing the head and tail. Phosphoric Acid, Urea, Ethanol, Sodium Chloride and other chemicals were all analytically pure and purchased from Sinopharm Chemical Reagent Co., Ltd. (Shanghai, China).

### 4.2. Preparation of Surimi and Crayfish Muscle Blended Gels

Crayfish muscle was chopped and mixed with surimi, and the amounts of crayfish muscle added were 0%, 2.5%, 5%, 7.5%, and 10%. Subsequently, salt (2.5%, *w*/*w*) and TGase (0.6%, *w*/*w*) were added, and chopping continued for 2 min. The homogeneous surimi mixture was then encased in sausage casings and sealed at both ends using a clipper. The encased surimi–crayfish mixtures underwent a two-stage heating process, being initially submerged in a 40 ± 2 °C water bath for 60 min, followed by undergoing gelation at 90 ± 2 °C for 30 min. The heat-treated sausages were subsequently cooled and stored overnight at 5 ± 3 °C.

### 4.3. Gel Strength

After reaching equilibrium at room temperature for approximately 60 min, the blended gels were sectioned into cylinders approximately 2 cm in height. Puncture tests were performed with a TA-XT Plus texture analyzer (Stable Micro Systems, Surrey, London, UK) fitted with a P/5S spherical probe, executing five replicates per sample [56]. Test settings were as follows: automatic trigger, trigger force = 5.0 g, both pretest and test speeds at 1.0 mm/s, post-test speed = 1.0 mm/s, pressing displacement = 15 mm, and a return speed of 10.0 mm/s. We calculated the gel strength according to Formula (1):

(1) Gel strength (g·cm)=Breaking force (g)×Deformation (cm)

### 4.4. WHC

Thin slices of approximately 5 g of the blended gel (2 mm) were wrapped in three layers of filter paper and centrifuged at 7040× *g* for 15 min at room temperature (Tianmei Scientific Instrument Co., Ltd., Zurich, Switzerland) [7]. We record the mass of the sample before (m1) and after (m2) centrifugation. Each sample was tested in triplicate, and then we calculated the WHC according to Formula (2):

(2) WHC (%)=(m2/m1)×100

### 4.5. Whiteness

Gel samples were sliced into cylinders about 10 mm thick, and their color attributes were measured using an automatic colorimeter (ZE7700, Nippon Denshoku Industries Co., Ltd., Tokyo, Japan) [7]. The measurements consisted of a* (red/green), b* (yellow/blue) and L* (luminance), and were performed six times for each sample. The whiteness was calculated according to Equation (3). Subsequently, ΔE was calculated to measure the difference in the overall color change in the blended gel before and after TGase addition under the addition of different amounts of crayfish. ΔE was calculated as shown in Equation (4):

(3) $$\mathrm{Whiteness}=100-\sqrt{{\left(100-L^{*}\right)}^{2}+{a^{*}}^{2}+{b^{*}}^{2}}$$

(4) $$\Delta E=\sqrt{({{L^{*}}_{2}-{L^{*}}_{1})}^{2}+({{a^{*}}_{2}-{a^{*}}_{1})}^{2}+({{b^{*}}_{2}-{b^{*}}_{1})}^{2}}$$

In Equation (4), L*_1_, a*_1_, and b*_1_ are the color characteristics of the blended gel without TGase with the same amounts of crayfish added, and L*_2_, a*_2_, and b*_2_ are the color characteristics of the blended gel with TGase and with the same amounts of crayfish added.

### 4.6. Low-Field Nuclear Magnetic Resonance

A low-field nuclear magnetic resonance analyzer (MesoMR23-060H-I, Niumag Electronic Technology Co., Ltd., Suzhou, China) was utilized to evaluate the water relaxation characteristics in surimi gel [2]. After removing the casing, 4 g of the sample was immediately transferred into a 15 mm diameter NMR tube for analysis. A CPMG sequence was utilized to capture transverse relaxation time (T2) signals at a resonance frequency of 18 MHz. The T2 relaxation data were processed through inversion, with each sample undergoing triple testing.

### 4.7. Magnetic Resonance Imaging

The proton density distribution in samples was analyzed using a multi-slice spin-echo pulse sequence and subsequently transformed into color images using pseudo-color processing [19]. The samples were sectioned into cylinders 20 mm in height and positioned within an NMR tube for imaging. The imaging parameters were set as follows: slice width = 3.0 mm, slice spacing = 1.0 mm, TE = 0.5 ms, and TR = 1800 ms.

### 4.8. SEM

The surface morphology of blended gel samples was examined. Gel samples were sectioned into 4 mm cubes and immersed in 0.1 M phosphate buffer (pH 7.2) for 20 min. Subsequently, the samples were stabilized in a 2.5% glutaraldehyde solution at 4 °C for a period of 24 h and rinsed five times with the same buffer. The samples then underwent a sequential dehydration process using ethanol solutions at concentrations increasing from 30% to 90% for 15 min each step, followed by dehydration in anhydrous ethanol for 20 min. After the removal of anhydrous ethanol, the samples underwent freeze-drying for a period of 24 h. The samples were surface sprayed with gold (Au-Pd alloy), and the surface features of the blended gels were observed with a scanning electron microscope (EM30+, COXEM Co., Ltd., Daejeon, Republic of Korea) at an operating voltage of 8 kV with a magnification of 2000 times [27]. After converting the raw SEM images into 8-bit grayscale images using ImageJ software (1.54f, National Institutes of Health, Bethesda, MD, USA), the “Threshold” tool (Otsu automatic thresholding) was used to distinguish between pore and matrix areas. By manually fine-tuning the threshold range (gray scale values 0–50 for pores and 51–255 for the matrix), it was ensured that the binarized image accurately reflected the actual pore structure [57]. Three porosity calculations were carried out as shown in Equation (5):

(5) Porosity (%)=(Pore area/Total image area)×100%

### 4.9. Chemical Interactions

Chemical interactions in the blended gels were analyzed by examining the differential solubility of chemical bonds in four different solutions: 0.05 M NaCl (SA), 0.6 M NaCl (SB), 0.6 M NaCl + 1.5 M urea (SC), and 0.6 M NaCl + 8 M urea (SD) [58]. In each experimental run, 10 mL of the designated solution was introduced to 2 g of finely minced gel samples, which were then homogenized for one minute. Subsequently, the mixture was then stirred gently at a steady 4 °C for 2 h before being centrifuged at 8000× *g* for 20 min. Using a detergent-compatible Bradford protein concentration assay kit (Beyotime Biotechnology Co., Ltd., Shanghai, China) to measure the protein content in the solution, absorbance at 595 nm^−1^ was measured with a microplate reader (Bio Tek Instruments Inc., Burlington, VT, USA), while protein concentrations were determined based on the formula provided with the kit. The quantification of ionic interactions (SB–SA), hydrogen bonding (SC–SB), and hydrophobic forces (SD–SC) was facilitated by the observed variations in protein concentration. Each measurement was replicated three times to ensure accuracy and consistency.

### 4.10. Total Sulfhydryl Content

An amount of 1 g of the blended gel sample was taken and mixed with 10 mL of the extraction solution and centrifuged at 8000× *g* for 10 min. The total sulfhydryl content in the supernatant was quantified using the sulfhydryl detection kit (Beijing Solarbio Science & Technology Co., Ltd., Beijing, China), adhering to the manufacturer’s guidelines. Sulfhydryl groups interact with DTNB to produce a yellow derivative that exhibits a prominent absorption peak at 412 nm^−1^. Absorbance at this wavelength was recorded using a microplate reader (Bio Tek Instruments Inc., Burlington, VT, USA), and the total sulfhydryl content was precisely calculated with the formula provided in the kit.

### 4.11. FTIR

Samples were sectioned into small fragments, freeze-dried under vacuum conditions, and then finely pulverized to minimize moisture interference during analysis. Spectral analysis was conducted using a Nicolet 6700 Fourier-transform infrared spectrometer (Thermo Fisher Scientific Inc., Waltham, MA, USA) [19]. Spectra were acquired from 500 to 4000 cm^−1^, with each measurement taken at a 4 cm^−1^ resolution. Air served as the background reference for calibration before experiments. The ATR crystal was cleaned with anhydrous ethanol before and after each measurement. Data accuracy was enhanced by averaging 32 scans. Using PeakFit 4.12 software, the Amide I band spectra (1600–1700 cm^−1^) were analyzed to ascertain the relative content of the secondary structure by deconvolution, Gaussian curve fitting, and second derivative calculations, represented as a percentage of the peak area.

### 4.12. Statistical Analysis

Data analysis was performed using SPSS 19.0 software. Data from blended gel samples with different contents of crayfish muscle were analyzed using one-way ANOVA. Data from two groups (without TGase and with TGase) of blended gel samples with the same contents of crayfish muscle were analyzed by independent *t*-tests. The results of data analysis were plotted graphically using Origin 2021 software.

## Figures and Tables

**Table 1:** The effect of TG on the chemical interactions in blended gels.

Crayfish Muscle Content (%)	Hydrogen Bonds		Ionic Bonds		Hydrophobic Interactions	
	Without TGase	With TGase	Without TGase	With TGase	Without TGase	With TGase
0	1.66 ± 0.04 ^Ba^	2.31 ± 0.12 ^Aa^	0.88 ± 0.03 ^Ae^	0.35 ± 0.04 ^Be^	21.99 ± 0.32 ^Aa^	20.1 ± 0.33 ^Bd^
2.5	1.27 ± 0.10 ^Bb^	2.09 ± 0.11 ^Ab^	1.25 ± 0.08 ^Ad^	0.66 ± 0.02 ^Bd^	20.21 ± 0.23 ^Bb^	21.1 ± 1.04 ^Ac^
5	0.97 ± 0.13 ^Bc^	1.74 ± 0.06 ^Ac^	1.60 ± 0.10 ^Ac^	0.95 ± 0.08 ^Bc^	18.1 ± 0.33 ^Bc^	22.82 ± 1.54 ^Ab^
7.5	0.47 ± 0.16 ^Bd^	1.37 ± 0.03 ^Ad^	2.04 ± 0.15 ^Ab^	1.27 ± 0.05 ^Bb^	16.55 ± 0.28 ^Bd^	25.6 ± 0.74 ^Aa^
10	0.21 ± 0.10 ^Be^	0.85 ± 0.05 ^Ae^	2.34 ± 0.12 ^Aa^	1.69 ± 0.03 ^Ba^	14.16 ± 0.9 ^Be^	25.62 ± 0.36 ^Aa^

Notes: Lowercase letters above each standard deviation bar reveal significant variations among gels with different crayfish muscle contents (*p* < 0.05). Uppercase letters highlight significant differences between samples with and without added TGase (*p* < 0.05).

## Data Availability

The original contributions presented in the study are included in the article, further inquiries can be directed to the corresponding author.
